# Deregulation of *ARID1A*, *CDH1*, *cMET* and *PIK3CA* and target-related microRNA expression in gastric cancer

**DOI:** 10.18632/oncotarget.4775

**Published:** 2015-07-27

**Authors:** Maider Ibarrola-Villava, Marta J. Llorca-Cardeñosa, Noelia Tarazona, Cristina Mongort, Tania Fleitas, José Alejandro Perez-Fidalgo, Susana Roselló, Samuel Navarro, Gloria Ribas, Andrés Cervantes

**Affiliations:** ^1^ Hematology and Medical Oncology Unit, Biomedical Research Institute INCLIVA, University of Valencia, 46010, Valencia, Spain; ^2^ Department of Pathology, Biomedical Research Institute INCLIVA, University of Valencia, 46010, Valencia, Spain

**Keywords:** gastric cancer, gene expression, microrna expression, immunohistochemistry, biomarkers

## Abstract

Genetic and epigenetic alterations play an important role in gastric cancer (GC) pathogenesis. Aberrations of the phosphatidylinositol-3-kinase signaling pathway are well described. However, emerging genes have been described such as, the chromatin remodeling gene *ARID1A*. Our aim was to determine the expression levels of four GC-related genes, *ARID1A*, *CDH1*, *cMET* and *PIK3CA*, and 14 target-related microRNAs (miRNAs).

We compared mRNA and miRNA expression levels among 66 gastric tumor and normal adjacent mucosa samples using quantitative real-time reverse transcription PCR. Moreover, ARID1A, cMET and PIK3CA protein levels were assessed by immunohistochemistry (IHC). Finally, gene and miRNAs associations with clinical characteristics and outcome were also evaluated.

An increased cMET and PIK3CA mRNA expression was found in 78.0% (*P* = 2.20 × 10^−5^) and 73.8% (*P* = 1.00 × 10^−3^) of the tumors, respectively. Moreover, IHC revealed that cMET and PIK3CA expression was positive in 63.6% and 87.8% of the tumors, respectively. Six miRNAs had significantly different expression between paired-samples, finding five up-regulated [miR-223-3p (*P* = 1.65 × 10^−6^), miR-19a-3p (*P* = 1.23 × 10^−4^), miR-128-3p (*P* = 3.49 × 10^−4^), miR-130b-3p (*P* = 1.00 × 10^−3^) and miR-34a-5p (*P* = 4.00 × 10^−3^)] and one down-regulated [miR-124-3p (*P* = 0.03)].

Our data suggest that *cMET*, *PIK3CA* and target-related miRNAs play an important role in GC and may serve as potential targets for therapy.

## INTRODUCTION

Cancer gastric (GC) is the third most common cause of cancer-related deaths [1] showing a higher incidence in Asian countries. However, the incidence of GC affecting some locations, particularly in the gastroesophageal junction, is rising in western countries. The aetio-pathogenesis of this disease remains unclear. Both environmental and genetic and epigenetic factors influence the development of the sporadic disease. Current treatments include surgery, chemotherapy and radiation therapy. However, prognosis for GC remains poor due to the absence of specific biomarkers for early detection, and lack of highly specific and effective therapies.

The traditional pathological classifications according to Lauren or World Health Organization have limited clinical utility. Recently, The Cancer Genome Atlas (TCGA) network has comprehensively characterized gastric adenocarcinomas and therefore proposed a new type of classification into four subtypes: Epstein-Barr virus positive, microsatellite instable, genomically stable and chromosomal instability tumors [2].

Our knowledge of GC has been reinforced by the development of high-throughput technologies such as next-generation sequencing or microarrays. Thus, comprehensive research into GC has allowed the identification of molecular targets in order to personalize treatments. Molecular profiling has become a useful tool in selecting personalized treatments for many solid tumors, whereas microarray technology has been widely used in functional genomics and systems biology.

In GC the most common recurrent genomic aberrations involve the *TP53*, *PIK3CA*, *ERBB2*, *ERBB3*, *ARID1A* and *KRAS* genes [3]. Other less frequent alterations included the *MET*, *FGFR1*, *MYC* and *CDH1* genes [4, 5]. Therefore many oncogenes and tumor suppressor genes have been related to GC. MicroRNAs (miRNAs) were also found to play an important role in GC [6, 7].

To assess their potential deregulation in GC, we studied *ARID1A*, *CDH1*, *cMET* and *PIK3CA* expression levels in gastric adenocarcinoma comparing them with normal gastric mucosa, using quantitative real-time reverse transcription PCR (RT-qPCR) and immunohistochemistry (IHC). We also determined 14 miRNAs expression levels. These miRNAs do target the analyzed genes.

## RESULTS

### Analyses of mRNA expression

**Figure 1:**
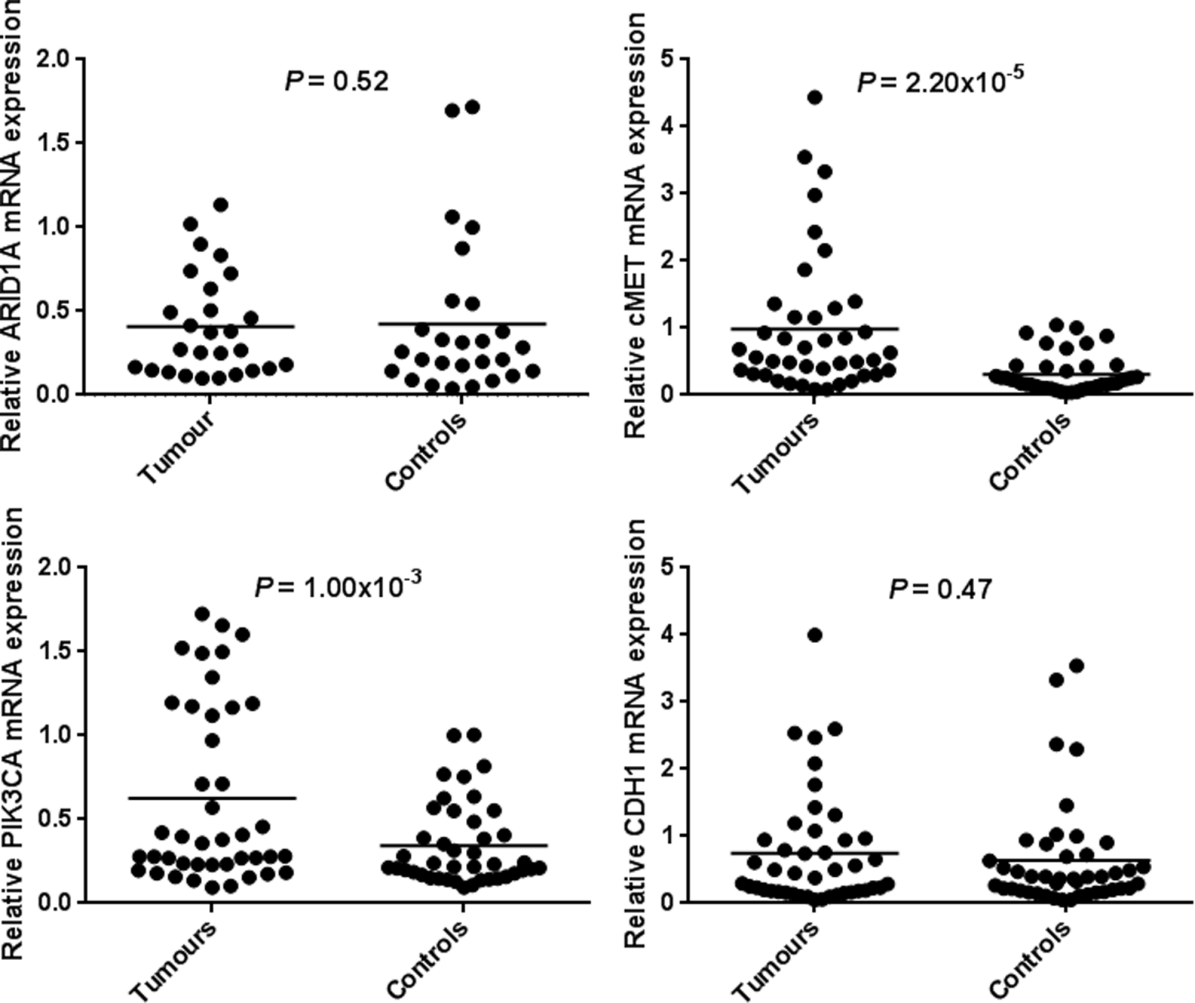
mRNA expression of ARID1A, CDH1, c-MET and PIK3CA in human gastric cancer tissues and paired-adjacent non-tumor gastric mucosa Expression analyses were determined by real-time quantitative PCR. Horizontal lines represent the mean. The relative mRNA expression of ARID1A was decreased in gastric tumor tissues whereas mRNA expression of CDH1, c-MET and PIK3CA was increased in gastric tumors tissues being significantly associated for the *c-MET* and *PIK3CA* genes.

Normal gastric mucosa and gastric tumor tissues were compared for ARID1A, CDH1, c-MET and PIK3CA mRNA expression using RT-qPCR. The c-MET and PIK3CA expression levels were significantly higher in 32 (78.0%) and 31 (73.8%) tumor tissues compared with adjacent non-tumor tissues (*P* = 2.20 × 10^−5^ and *P* = 1.00 × 10^−3^ respectively). Similarly, CDH1 expression was slightly higher in 24 (54.5%) tumor samples although no statistical difference was found (*P* = 0.47). Although a decreased ARID1A expression was detected in tumor tissues, it did not remain statistically significant (*P* = 0.52). Gene expression results are shown in Table 1 and Figure 1. None correlation was observed between cMET and PIK3CA mRNA expression among tumors samples (*r* = − 0.12, *P* = 0.95).

**Table 1:** Levels of expression of the *ARID1A*, *CDH1*, *cMET* and *PIK3CA* genes among tumour samples and their adjacent normal-paired tissue

Gene	*N*	Tumour Samples Percentiles			Control Samples Percentiles			*P*
		25	50	75	25	50	75	
*ARID1A*	27	0.15	0.27	0.64	0.14	0.26	0.55	0.524
*CDH1*	44	0.18	0.42	0.96	0.19	0.38	0.72	0.472
*cMET*	41	0.31	0.56	1.23	0.10	0.22	0.42	**2.20 × 10^−5^**
*PIK3CA*	42	0.23	0.37	1.17	0.17	0.23	0.50	**1.00 × 10^−3^**

*N*, number of control-paired samples

*P* value obtained according to the Wilcoxon rank test. Normality test was performed and *P* values were < 0.01

Bold indicates significant results

### Analyses of protein expression

**Figure 2:**
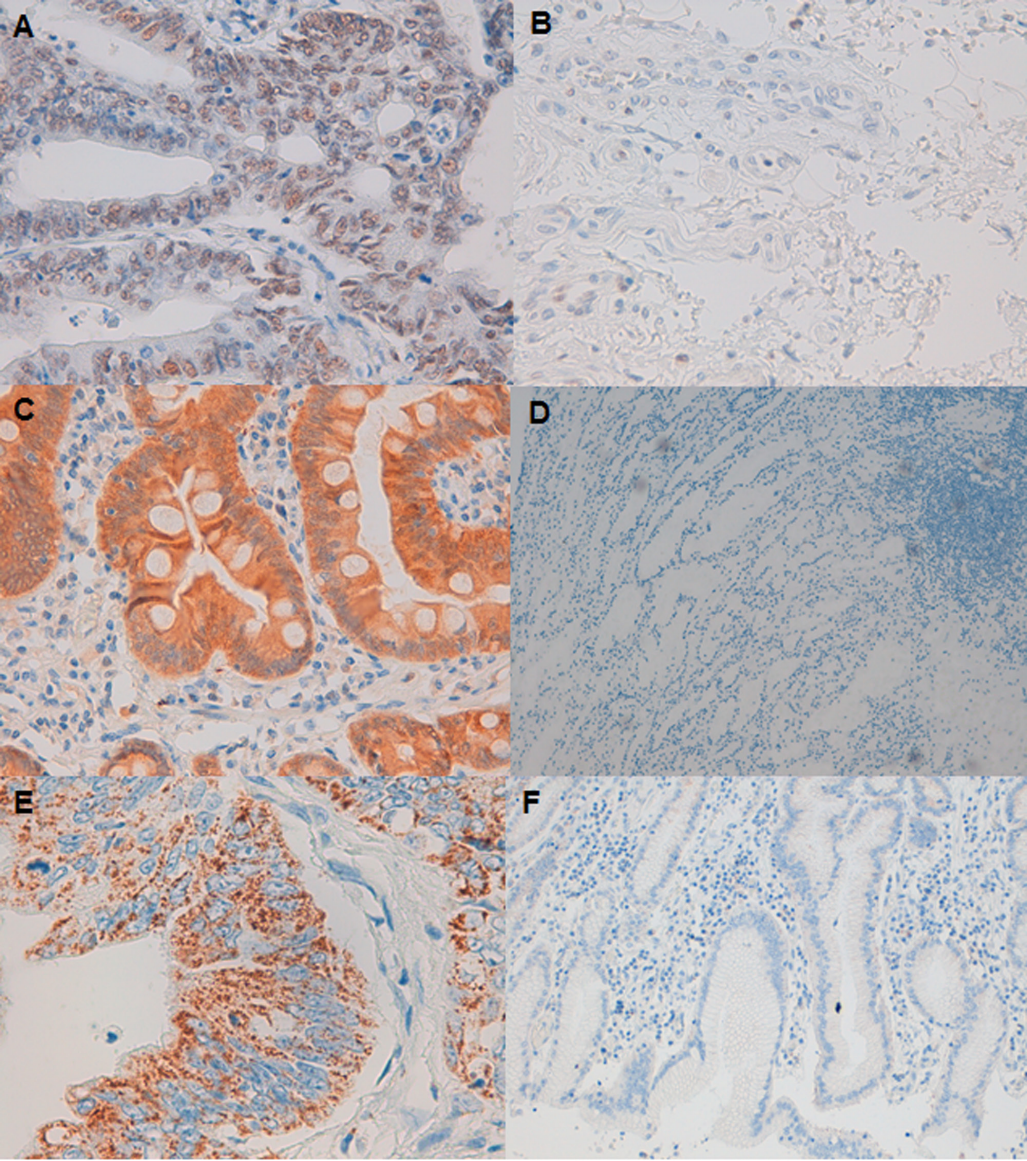
ARID1A, c-MET and PIK3CA protein expression in gastric cancer tumor shown by immunohistochemistry **A.** Strong nuclear ARID1A staining (original magnification: 40X). **B.** ARID1A-negative staining (40X). **C.** Positive cytoplasmic expression of cMET (40X). **D.** Negative expression of cMET (10X). **E.** PIK3CA granular cytoplasm positive staining (40X). **F.** PIK3CA negative staining (10X).

To further investigate the role of *ARID1A* in GC, we performed IHC analysis of ARID1A. Furthermore, the expression and location of c-MET and PIK3CA proteins were also studied by IHC in resected tumor tissues. A total of 33 paired-samples were analyzed. When comparing protein expression with their paired-control sample, ARID1A levels were lower in 42.4% of the tumor tissues whereas c-MET and PIK3CA levels were higher in 36.4% and 63.6% of the tumor tissues analyzed (Figure 2). Among tumor samples, ARID1A expression was negative in 27.3% of the tissues whereas cMET and PIK3CA expression was positive in 63.6% and 87.8% of the tumors respectively (Table 2).

**Table 2:** Levels of expression of the ARID1A, cMET and PIK3CA proteins among tumour samples and their adjacent normal-paired tissue by immunohistochemistry analysis

**a. ARID1A**	**Control**			
Tumour	Negative	Positive	Total	*P* value
Negative	1	7	8	0.08
Positive	0	24	24	
Total	1	31	32	
**b. cMET**	**Control**			
Tumour	Negative	Positive	Total	*P* value
Negative	1	11	12	0.18
Positive	6	15	21	
Total	7	26	33	
**c. PIK3CA**	**Control**			
Tumour	Negative	Positive	Total	*P* value
Negative	0	4	4	0.07
Positive	14	15	29	
Total	14	19	33	

*P* value according to Pearson correlation test

### Analyses of miRNA expression

**Figure 3:**
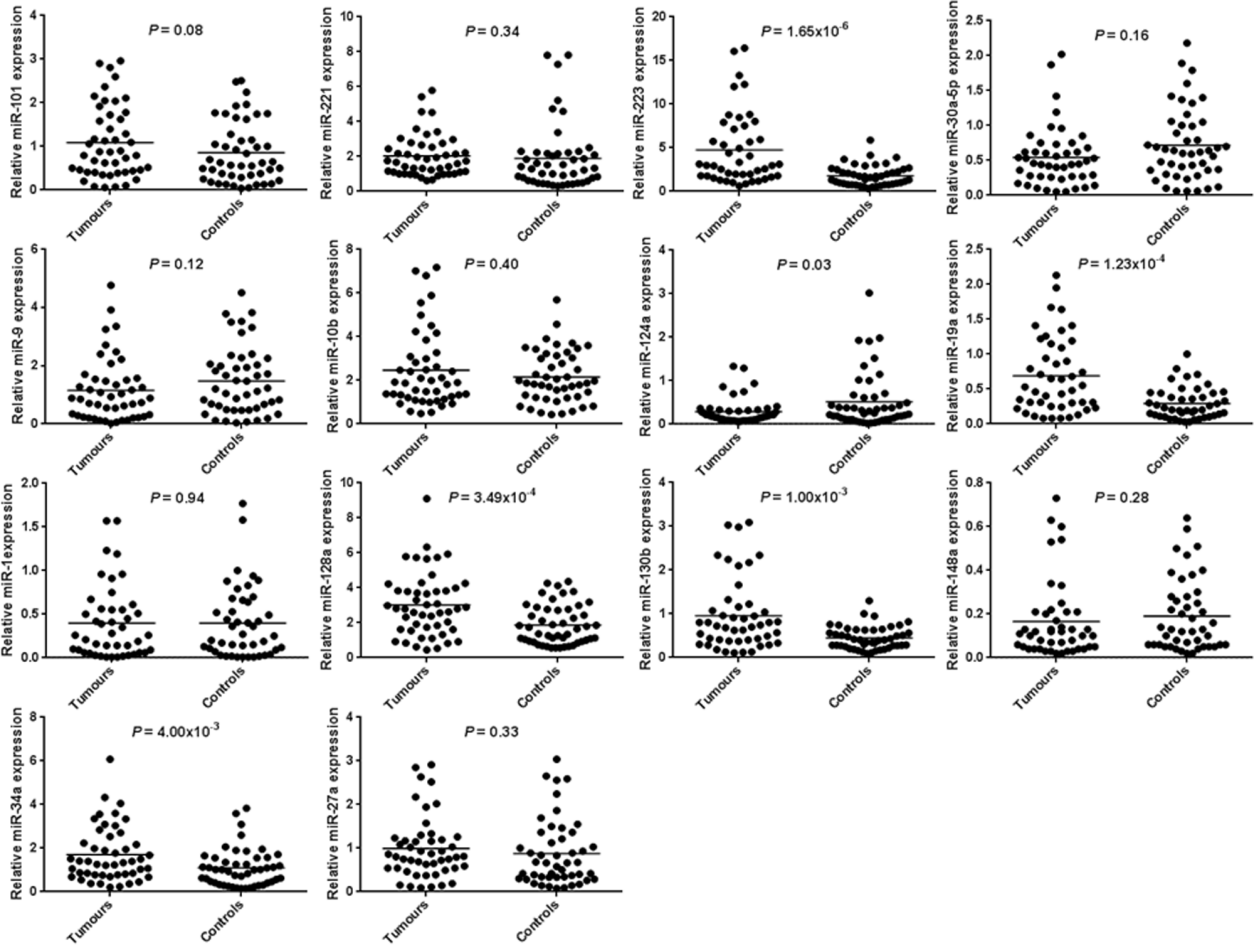
microRNA expression of 14 microRNAs in human gastric cancer tissues and paired-adjacent non-tumor gastric mucosa Expression analyses were determined by real-time quantitative PCR. Horizontal lines represent the mean.

Normal gastric mucosa and gastric tumor tissues were compared for 14 miRNAs expression using RT-qPCR. Six miRNAs had significantly different expression between paired-samples. The miR-223-3p, miR-19a-3p, miR-128-3p, miR-130b-3p and miR-34a-5p expression levels were significantly higher in 38 (84.4%), 31 (68.9%), 35 (71.4%), 31 (66.0%) and 35 (72.9%) tumor tissues compared with adjacent non-tumor tissues (*P* = 1.65 × 10^−6^, *P* = 1.23 × 10^−4^, *P* = 3.49 × 10^−4^, *P* = 1.00 × 10^−3^ and *P* = 4.00 × 10^−3^ respectively). The miR-124-3p expression level was significantly lower in 26 tumor tissues compared with adjacent non-tumor tissues (54.2%, *P* = 0.03). MiRNA expression results and target-related gene are shown in Table 3 and Figure 3. None significant correlation was observed when *PIK3CA* miR-19a-3p and miR-124-3p target miRNAs are compared (*r* = 0.22, *P* = 0.17). However, when *cMET* miR-128-3p and miR-34a-5p, miR-128-3p and miR-130b-3p and miR-130b-3p and miR-34a-5p were compared we observed significant associations (*r* = 0.33, *P* = 0.03; *r* = 0.64, *P* = 3.63 × 10^−6^; *r* = 0.36, *P* = 0.02 respectively). *cMET* significantly associated miRNAs were subjected to a principal component analysis which revealed that miR-128-3p, miR-130b-3p and miR-34a-5p expressions are similar and construct a unique component (*KMO* = 0.61 and *P* value of Bartlett = 1.22 × 10^−4^).

**Table 3:** Levels of expression of microRNAs among tumour samples and their adjacent normal-paired tissue

Target Gene	miRNA	*N*	Tumour samples percentiles			Control samples percentiles			*P* value
			25	50	75	25	50	75	
*ARID1A*	miRNA-101–3p^¥^	47	0.41	0.87	1.72	0.29	0.62	1.28	0.11
	miRNA-221–3p	47	1.10	1.74	2.65	0.59	1.33	2.22	0.34
	miRNA-223–3p	45	1.77	2.98	7.32	0.81	1.59	2.50	1.65 × 10^−6^*
	miRNA-30a-5p	46	0.27	0.46	0.69	0.34	0.63	1.01	0.16
*CDH1*	miRNA-9–5p^¥^	47	0.25	0.89	1.54	0.49	1.20	2.06	0.15
*cMET*	miRNA-1–3p^¥^	44	0.06	0.26	0.56	0.08	0.26	0.66	0.99
	miRNA-128–3p^¥^	49	1.62	2.80	3.91	0.94	1.65	2.76	1.85 × 10^−4^*
	miRNA-130b-3p	47	0.38	0.72	1.16	0.27	0.40	0.63	1.00 × 10^−3^*
	miRNA-148a-3p	45	0.04	0.11	0.21	0.06	0.13	0.28	0.28
	miRNA-27a-3p^¥^	48	0.54	0.80	1.22	0.33	0.66	1.32	0.42
	miRNA-34a-5p	48	0.78	1.64	2.46	0.40	1.00	1.55	**4.00 × 10^−3^**
*PIK3CA*	miRNA-10b-5p^¥^	47	1.20	1.90	3.09	1.21	1.88	3.13	0.26
	miRNA-124–3p	48	0.09	1.18	0.33	0.12	0.28	0.62	**0.03**
	miRNA-19a-3p^¥^	45	0.24	0.53	1.12	0.12	0.20	0.44	1.76 × 10^−5^*

*N*, number of control-paired samples

*P* value obtained according to the Wilcoxon rank test.

¥ *P* value obtained according to *t*-test

Bold indicates significant results

* Results significant after adjusting by Bonferroni

### Pathway enrichment

Kyoto Encyclopedia of Genes and Genomes (KEGG) pathway enrichment analysis revealed several pathways overrepresented with FDR *P* values < 0.05. These pathways included biological proliferation or differentiation related pathways relevant in cancer such as ErbB, mTOR, WNT, TGFβ, MAPK or PI3K/AKT signaling pathways, involved in melanogenesis, colorectal, prostate, endometrial or non-small cell lung cancer. Adhesion and mobility related pathways are also represented such as gap junction and focal adhesion.

### Association with clinical characteristics

We assessed whether these different gene and miRNAs expression levels were associated with various clinical characteristics. We observed evidence of association for less *CDH1* expression and diffuse type, according Lauren classification (*P* = 0.014). We also observed evidence of association for low *PIK3CA* expression and higher clinical stage (*P* = 0.024). Among miRNAs, miR-9-5p was associated with older age (*P* = 0.03), miR-221-3p was associated with tumor body or antrum location (*P* = 0.02), miR-128-3p and miR-130b-3p were associated with higher clinical stage (*P* = 0.002 and *P* = 0.003 respectively) and miR-19a-3p and miR-130b-3p were associated with microsatellite instability (*P* = 0.02 and *P* = 0.04 respectively). None of the other associations remained significant (Supplementary Table S2).

### Gene expression and clinical outcome

The overall survival of patients with negative *ARID1A* expression was significantly better than that of *ARID1A* positive patients (*P* = 0.03, log rank test, Supplementary Figure S1). There were no significant survival differences for *CDH1*, *cMET* and *PIK3CA* expression (*P* = 0.09, 0.92 and 0.94 respectively).

## DISCUSSION

Understanding the roles that genetic and epigenetic alterations play in GC pathogenesis has increased over the last decade. Recent next generation sequencing studies have identified recurrent somatic mutations in a number of potential cancer genes, such as the chromatin remodeling genes *ARID1A*, *MLL3* and *MLL*, the cell adhesion gene *FAT4*, the lipid kinase *PIK3CA* and the *P53* tumor-suppressor gene. Epigenetic regulation is essential for the normal development and maintenance of tissue-specific gene expression patterns. Therefore, disruption of epigenetic regulation can lead to aberrant gene function and malignant cellular transformation [8]. Recent studies of GC epigenetics have revealed widespread miRNAs alterations. MiRNAs are endogenous, small non-coding RNAs, 18–25 nucleotides in length that regulates gene expression at the posttranscriptional level through binding to target mRNAs. It is well known that miRNAs have important regulatory functions in biological processes such as development, cellular differentiation, proliferation, apoptosis and cancer [9, 10].

We aimed to identify four candidate differentially expressed mRNAs: ARID1A, CDH1, cMET and PIK3CA and 14 miRNAs that target these genes in GC.

The *PIK3CA* gene is located on chromosome 3p26.3 and encodes the key enzymatic subunit p110α of PI3K [11]. Mutations of the *PIK3CA* gene are highly prevalent in a variety of human solid tumors including colon, gastric and breast cancers [12, 13] and lead to dysregulation of PI3K/AKT signaling pathway [14]. Therefore, *PIK3CA* is thought to act as an oncogene. Gain of function *PIK3CA* mutations are frequently found in exons 9 and 20 including different hotspot mutations [13] and PI3K inhibitors for GC are currently in clinical testing [15]. However, the relationship between mRNA PIK3CA expression and miRNAs targeting this gene and GC has been rarely reported.

The *c-MET* gene is located in chromosome 7q31 and is a proto-oncogene that encodes a transmembrane high affinity tyrosine kinase receptor for hepatocyte growth hormone. Overexpression of *c-MET* has been described in many tumor types such as gastric, oesophageal or lung [16, 17]. The *MET* gene can also carry activating mutations although are exceedingly rare in GC [18, 19]. MET inhibitors have been introduced into the clinical application although conflicting results have been reported [20].

The c-MET and PIK3CA mRNA expression levels were independently significantly higher in tumor tissues compared with adjacent non-tumor tissues and these results were confirmed by IHC. PIK3CA mRNA and protein expression were reported to be significantly higher in gastric tumor tissues compared to normal gastric adjacent mucosa [12, 21, 22]. Furthermore, PIK3CA overexpression was described in other different tumor types [23, 24]. Regarding clinical associations, PIK3CA gain was significantly associated with lower clinical stage. Other clinicopathological factors such as age, gender or tumor location were statistically irrelevant to the positive expression of PIK3CA as previously reported [21]. The increased expression of PIK3CA did not affect the prognosis of GC patients [25]. Increased cMET mRNA and protein expression detected in our GC series has been confirmed by others [26, 27]. cMET gain was not significantly associated with any clinical characteristics or a better overall survival.

The *CDH1* gene is located in chromosome 16q22.1 and encodes E-cadherin, a member of the cadherin superfamily of calcium-dependent cell adhesion molecules. E-cadherin has a well-documented role in the progression of epithelial cancers. Inactivating mutations in the *CDH1* gene are frequently found in GC, especially in hereditary diffuse GC [28]. *CDH1* promoter methylation is also frequently found in sporadic GC [29]. The down-regulation of the protein during carcinoma invasion and metastasis has led to the concept that E-cadherin acts as a tumor suppressor gene during epithelial tumorigenesis [30]. However, the role of CDH1 mRNA expression has been barely documented. In our study *CDH1* expression was not statistical difference between tumor gastric and normal mucosa control samples. Conflicting results have been reported among different tumor types [31, 32]. We found an association between CDH1 high expression levels and intestinal gastric subtypes. No further association was found with survival.

The *ARID1A* gene is located in chromosome 1p35.3 and encodes one of the subunits of the Swith/Sucrose Non-Fermentable chromatin remodeling complex. *ARID1A* is highly mutated in tumors and has recently been identified as a novel tumor suppressor in different cancer types [33, 34]. ARID1A mRNA and protein expression were reported to be significantly lower in gastric tumor tissues compared to normal gastric adjacent mucosa [33]. In our study, *ARID1A* expression was lower in tumor samples compared to control samples although no statistical differences were found. However, a considerable number of samples failed for *ARID1A* RT-qPCR expression. RT-qPCR expression probe is designed at the beginning of exon 5. To further investigate the role of *ARID1A* in GC, we performed IHC analysis of ARID1A in 33 paired-tumor samples. Protein levels were lower in 40.6% of the tumors when comparing with their adjacent normal mucosa. Furthermore, when considering only tumor samples, 27.3% of them had negative expression, which is in accordance with other published results [35]. We can think that those negative tumor samples for both gene and protein expression have a deletion which makes impossible to obtained results. ARID1A loss was not significantly associated with any clinical characteristics as reported by others [35]. Neither was associated with a better survival of the patients as previously reported [36, 37].

In order to generate combined biomarkers to be used for early diagnosis of GC or prediction of survival and treatment responses, the role of 14 different miRNAs expression was compared between tumor tissues and normal gastric mucosa. Four miRNAs were selected for the *ARID1A* gene, one for the *CDH1* gene, 6 for the *cMET* gene and 3 for the *PIK3CA* gene. Six miRNAs had significantly different expression between paired-samples. The miR-223-3p, miR-19a-3p, miR-128-3p, miR-130b-3p and miR-34a-5p expression levels were significantly higher in tumor tissues compared with adjacent non-tumor tissues. The miR-124-3p expression level was significantly lower in tumor tissues compared with adjacent non-tumor tissues.

MiR-223-3p was selected due to potential binding in the 3′UTR of the ARID1A mRNA and high expression levels were previously reported in GC [38–40]. Furthermore, miR-223-3p expression was significantly higher in oesophageal cancer tissues than in the corresponding normal mucosa [41]. MiR-19a-3p and miR-124-3p were selected due to potential target of the *PIK3CA* gene. MiR-19a-3p and miR-124-3p expression were independently associated to GC. MiR-19a-3p was consistently reported upregulated and it is described to promote epithelial-mesenchymal transition through PI3K/AKT pathway in GC [40, 42]. However, miR-124-3p was reported to be down-regulated in many different cancer types, including gastric tumors, where proliferation resulted inhibited [43, 44]. Finally, miR-128-3p, miR-130b-3p and miR-34a-5p were selected due to cMET 3′UTR putative binding and their expressions were similar. For these three miRNAs high expression levels have been described in GC [38, 45].

Finally some association between miRNAs and clinical characteristics were found. To the best of our knowledge the majority of these associations are described for the first time in GC. Firstly, miR-9-5p was associated with older age. Secondly, miR-221-3p was associated with body or antrum tumor location. This miRNA was previously associated with tumor-node-metastasis stage [46, 47]. Moreover, miR-19a-3p and miR-130b-3p were associated with MSI. Furthermore, miR-130b-3p was associated with higher clinical stage as previously described [45]. MiR-128-3p was also associated with higher clinical stage.

In conclusion, we have identified two genes, *cMET* and *PIK3CA*, and 6 miRNAs, miR-223-3p, miR-19a-3p, miR-128-3p, miR-130b-3p, miR-34a-5p and miR-124-3p that were statistically differently expressed in gastric tumor tissues compared with normal mucosa. Furthermore, some clinicopathological associations have also been described in GC.

One of the main limitations of our study is its exploratory design. There is a need for a better understanding of the molecular features, which characterize the different subtypes of gastric cancer. What we describe in our article is a differential deregulation of some genes involved in gastric cancer pathogenesis, as well as some related miRNAs. However, before applying them in clinical practice a deeper analysis and validation in prospective trial is needed.

## MATERIALS AND METHODS

### Patient selection and data collection

A total of 82 paired tumor and matched adjacent non-cancerous gastric mucosa tissues were recruited from GC patients between January 2013 to December 2013 at the Hematology and Medical Oncology Unit of the Biomedical Research Institute INCLIVA in Valencia, Spain. Patient eligibility criteria included consecutive and non-related cases. According to clinical stage, patients at early stages (I-IIA) were subjected to surgery whereas patients at late stages (IIB-IV) were candidates or not for chemotherapy with/without radiotherapy and with/without gastrectomy. Clinic information, including age, sex, tumor location, microsatellite status and treatments were collected (See Supplementary Table 1). All study subjects gave written informed consent, and the study was approved by the Biomedical Research Institute INCLIVA Ethics Board.

Formalin-fixed paraffin-embedded (FFPE) tissues were evaluated for their tumor content and sections containing more than 30% of tumor cells were defined and cut by a pathologist. RNA was isolated from 4 unstained sections of 20 μm. This was done using Recover All Total Nucleic Acid Isolation kit (Ambiom, Life Technologies, Austin, TX, USA). RNA concentration was quantified in samples by NanoDrop (NanoDrop Technologies, Wilmington, DE, USA). Due to insufficient amount or quality of RNA, 16 patients were excluded from the paired analyses and therefore only 66 patients were included in the study. RNA samples were immediately frozen and stored at −80°C.

### Real-time quantitative PCR for gene expression

ARID1A, CDH1, c-MET and PIK3CA mRNA expression was analyzed by RT-qPCR on total RNA isolated from normal gastric mucosa and gastric tumor tissues.

Reverse transcription was performed with 200 ng of RNA in a total volume of 10 μl using the High Capacity cDNA Transcriptase Reverse kit (Applied Biosystems by Life Technologies, Carlsbad, California, USA) according to manufacturer's recommendations. Reaction was performed on a 96 thermal cycler with the following profile: 10 min at 25°C, 2 h at 37°C and 5 min at 85°C. A total of 2.5 μl of the resulting cDNA was subjected to pre-amplification using the TaqMan Pre-Amp Master Mix (Applied Biosystems) in a total volume of 12 μl. Non-fluorescent probes were used at 1X. Pre-amplification cycling conditions were 10 min at 95°C followed by 14 cycles, each one consisting of 15 s at 95°C and 4 min at 60°C. Later on a 1:5 dilution of the pre-amplified cDNA was performed. RT-qPCR was performed on the 7900HT Fast Real-Time PCR system using TaqMan gene expression assays probes (Applied Biosystems). The assay identification numbers were Hs00195464_m1 for ARID1A, Hs01023894_m1 for CDH1, Hs01565584_m1 for c-MET and Hs00907957_m1 for PIK3CA. Human glyceraldehyde-3-phosphate dehydrogenase, *GADPH* (Hs03929097_g1), was used as an internal control. The PCR was carried out in a total volume of 10 μl containing 1.5 μl of diluted and pre-amplified cDNA, 10 μl of TaqMan Gene Expression Master Mix and 1 μl of each fluorescence TaqMan probe. The cycling conditions were 50°C for 2 min, 95°C for 10 min followed by 40 cycles, each one consisting of 15 s at 95°C and 1 min at 60°C. Samples were run in triplicate and the mean value was calculated for each case.

The data were managed using the Applied Biosystems software RQ Manager v1.2.1. Relative expression was calculated by using comparative Ct method and obtaining the fold change value (2^−ΔΔCt^) according to previously described protocol [48].

### Immunohistochemistry

Three different primary antibodies were analyzed: a polyclonal rabbit antibody against ARID1A (HPA005456, dilution 1:500, Sigma-Aldrich), a polyclonal rabbit antibody against PIK3CA (HPA009985, dilution 1:200, Sigma-Aldrich) and a monoclonal rabbit antibody against cMET (Dako, Glostrup, Denmark).

The 2 μm tissue sections were cut into coated slides, deparaffinised with xilol and rehydrated through 90%, 80% and 70% ethanol. After washing in water, the slides were autoclaved for 3 min at 1.5 atmospheres in sodium citrate buffer (pH = 6 for ARID1A and PIK3CA and pH = 9 for cMET) for antigen retrieval. Endogenous peroxidase activity was blocked with hydrogen peroxidase for 5 min at room temperature. After rinsing with tris buffered saline 1X (TBS), the tissue sections were incubated with primary antibodies for 30 min. The sections were subsequently washed with TBS 1X and incubated with secondary antibody for 30 min for ARID1A and PIK3CA (K5007, DakoReal™ EnVision™ HRP Rabbit/Mouse, Dako, Glostrup, Denmark). For cMET preparations were incubated for 60 min and latter incubated with another antibody for 30 min (EnVision™ FLEX anti rabbit, Dako, Glostrup, Denmark). Diaminobenzidine (DAB) and haematoxylin chromogen (Dako, Glostrup, Denmark) method was used. The sections were subsequently examined by light microscopy and the intensity of staining was relative qualified (− = negative, + = low < 25%, ++ = medium 50%, +++ = high 100%).

Tumors were regarded as positive for ARID1A if tumor cells showed nuclear inmunoreactivity. However, for cMET and PIK3CA, when tumor cells showed cytoplasm inmunoreactivity. PIK3CA showed granular cytoplasm inmunoreactivity. Non-neoplastic cells, such as fibroblasts, endothelial cells, and lymphocytes, served as internal positive controls.

### Real-time quantitative PCR for miRNA expression

Fourteen different miRNAs targeting the above mentioned genes were selected and analyzed by RT-qPCR on total RNA isolated from normal gastric mucosa and gastric tumour tissues. Reverse transcription using the microRNA reverse transcription kit (Applied Biosystems by Life Technologies, Carlsbad, California, USA) and cDNA pre-amplification, were performed as described for mRNA expression. Subsequent dilution of pre-amplified cDNA was 1:10. RT-qPCR was performed using TaqMan microRNA Assays (Applied Biosystems). The assay identification numbers will be given upon request. Normalization was done with RNU6B miRNA. The PCR was carried out in a total volume of 10 μl containing 1.5 μl of diluted and pre-amplified cDNA, 10 μl of TaqMan Gene Expression Master Mix and 1 μl of each fluorescence probe. The cycling conditions were 50°C for 2 min, 95°C for 10 min followed by 45 cycles, each one consisting of 15 s at 95°C and 1 min at 60°C. Samples were run in triplicate and the mean value was calculated for each case.

The data were managed according to previously described protocol [48].

### Pathway enrichment analysis and candidate gene searching

Target-scan online software and previous literature were used to select 14 miRNAs among the four candidate genes (http://www.targetscan.org/vert_61/).

DIANA miRPath pathway enrichment analysis was used to gain insight into global molecular networks and canonical pathways related to differentially expressed miRNAs (http://diana.imis.athena-innovation.gr/DianaTools/index.php?r=mirpath/index). The software performs an enrichment analysis of multiple miRNA target genes comparing each set of miRNA targets to all known Kyoto Encyclopedia of Genes and Genomes (KEGG) pathways (http://www.genome.jp/kegg/pathway.html). Those pathways showing a FDR *p*-value < 0.05 were considered significantly enriched.

### Statistical analysis

Differences in the mRNA expression results were analyzed by the Wilcoxon rank test after normalization testing. Differences in the miRNA expression results were analyzed by both the *t*-test and the Wilcoxon rank test after normalization testing. Correlation analyses were performed using the Pearson correlation test and the principal component analysis. Firstly, a Pearson correlation test was performed for those differently expressed genes. Secondly, another Pearson correlation test was performed for those significantly associated miRNAs. Association analyses for both mRNA and miRNAs expression with clinical characteristics, were performed using the Mann-Whitney U test after normalization testing. Survival curves were calculated using the Kaplan-Meier method and compared by the log-rank test according to univariate analysis. Analyses were performed using SPSS v19.0 (SPSS, Chicago, IL, USA) and two-sided *P* value less than 0.05 was considered to be statistically significant. SPSS v19.0 and GraphPad Prism v6.0 (GraphPad Software Inc., CA, USA) were used to depicting the results.

## SUPPLEMENTARY FIGURE AND TABLES


