# Supplementary material for: Deregulation of ARID1A, CDH1, cMET and PIK3CA and target-related microRNA expression in gastric cancer
Source: Oncotarget. 2015 Jul 27;6(29):26935–45. doi: 10.18632/oncotarget.4775 (PMC4694964; doi:10.18632/oncotarget.4775)
Supplement: Supplementary file 1 [file oncotarget-06-26935-s001.pdf]

SUPPLEMENTARY FIGURE AND TABLES

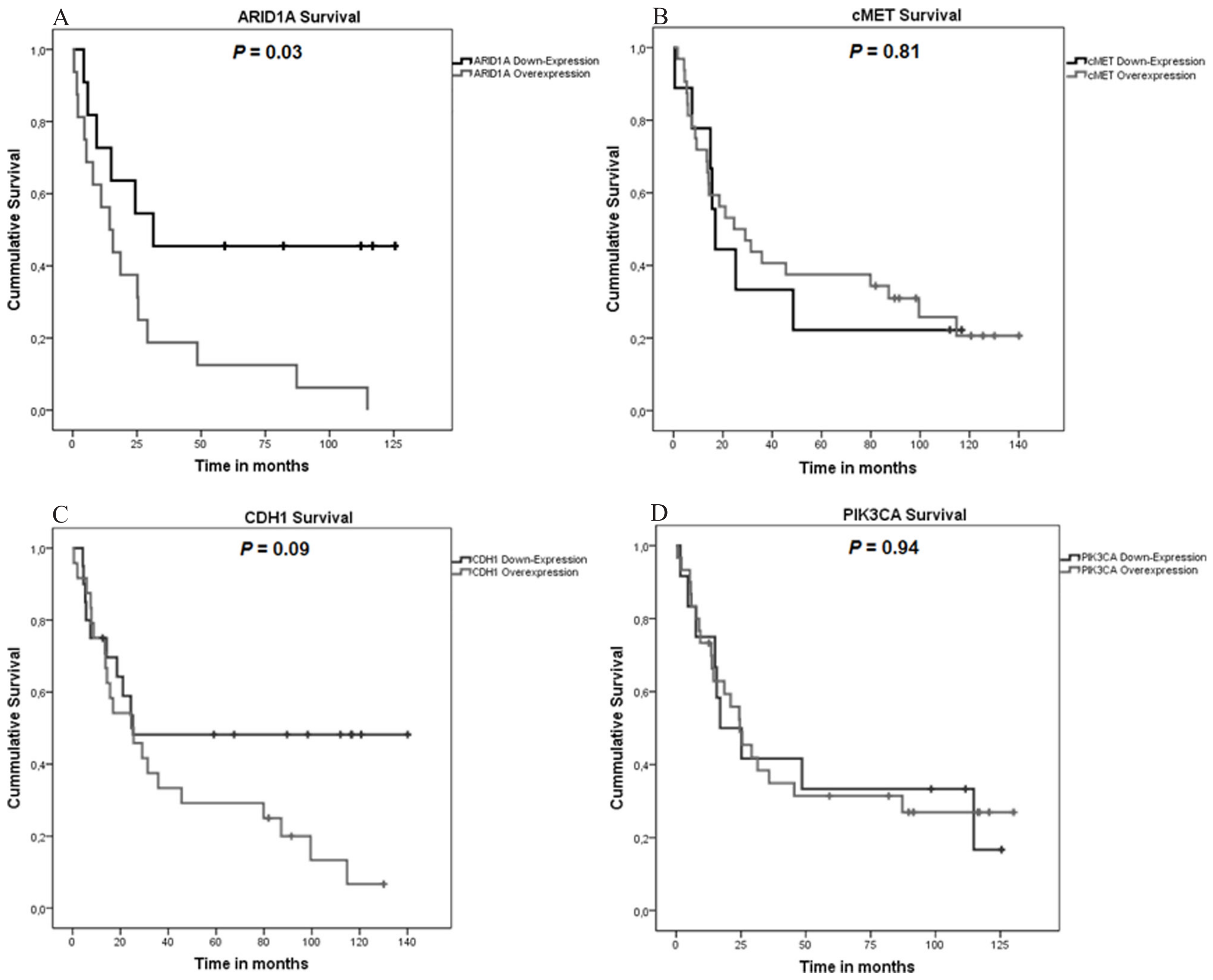

Supplementary Figure S1: Kaplan-Meier survival curves of gastric cancer patients according to univariate analysis. A. Patients with down- or up-expression of ARID1A. **B.** Patients with down- or up-expression of cMET. **C.** Patients with down- or up-expression of CDH1. **D.** Patients with down- or up-expression of PIK3CA.

Supplementary Table S1: Clinical characteristics of gastric cancer samples

Clinical characteristics		Number of samples *N* (%)
Age (years)	≤55	12 (18.2)
	>55	54 (81.8)
	Mean (SD)	67.4 (12.3)
Gender	Female	22 (33.3)
	Male	44 (66.7)
Tumour location	Antrum – Body	45 (68.2)
	Fundus – Cardias – Oesophageal union	20 (30.3)
	Gastric stump	1 (1.5)
Clinical stage	I-II-III	55 (83.3)
	IV	9 (13.6)
	Unknown	2 (3.0)
Pathological stage	I	8 (12.1)
	II	18 (27.3)
	III	27 (40.9)
	IVA	4 (6.1)
	Not determined	9 (13.6)
Lauren classification	Diffuse	24 (36.4)
	Intestinal	36 (54.5)
	Mixed	1 (1.5)
	Unknown	5 (7.6)
Histological grade	I	5 (7.6)
	II	14 (21.2)
	III	20 (30.3)
	Unknown	27 (40.9)
Microsatellite stability	Stable	49 (74.2)
	Unstable	10 (15.2)
	Unknown	7 (10.6)
Relapse	Yes	25 (37.9)
	No	33 (50.0)
	Unknown	8 (12.1)
Status	Alive	21 (31.8)
	Exitus	41 (62.1)
	Unknown	2 (3.0)

SD. Standard deviation

Supplementary Table S2A: Association between gene and microRNA expression and clinic characteristics

Expression	*ARID1A*	*CDH1*	*cMET*	*PIK3CA*	miR-1-3p	miR-9-5p	miR-10b-5p	miR-19a-3p	miR-27a-3p
**Clinic Characteristic**	***P***	***P***	***P***	***P***	***P***	***P***	***P***	***P***	***P***
Age	0.25	0.25	0.49	0.53	0.14	**0.03**	0.48	0.65	0.21
Gender	0.05	0.81	0.50	0.17	0.75	0.55	0.48	0.75	0.49
Tumour location	0.08	0.26	0.44	0.20	0.66	0.71	0.10	0.29	0.78
Clinical Stage	0.82	0.11	0.21	**0.02**	0.53	0.41	0.73	0.78	0.38
Lauren Classification	0.50	**0.01**	0.43	0.76	0.13	0.06	0.27	0.92	0.06
Microsatellite status	0.60	0.58	0.85	0.48	0.44	0.38	0.82	**0.02**	0.59

Bold represents significant results

Supplementary Table S2B

Expression	miR-30a-5p	miR-34a-5p	miR-101-3p	miR-124-3p	miR-128-3p	miR-130b-3p	miR-148a-3p	miR-221-3p	miR-223-3p
**Clinic Characteristic**	***P***	***P***	***P***	***P***	***P***	***P***	***P***	***P***	***P***
Age	0.08	0.33	0.66	0.29	0.79	0.17	0.93	0.08	0.34
Gender	0.44	0.18	0.14	0.93	0.59	0.60	0.84	0.53	0.12
Tumour location	0.98	0.68	0.63	0.91	0.78	0.84	0.38	**0.02**	0.31
Clinical Stage	0.70	0.36	0.15	0.36	**0.002**	**0.003**	0.33	0.51	0.35
Lauren Classification	0.12	0.08	0.50	0.11	0.22	0.63	0.96	0.39	0.51
Microsatellite status	0.80	0.62	0.11	0.80	0.86	**0.04**	0.61	0.52	0.55

Bold represents significant results
